# Positive Emotions Program for Schizophrenia (PEPS): a pilot intervention to reduce anhedonia and apathy

**DOI:** 10.1186/s12888-015-0610-y

**Published:** 2015-09-29

**Authors:** Jérôme Favrod, Alexandra Nguyen, Caroline Fankhauser, Alban Ismailaj, Jean-David Hasler, Abel Ringuet, Shyhrete Rexhaj, Charles Bonsack

**Affiliations:** 1 School of nursing sciences, La Source, University of Applied Sciences and Arts of Western Switzerland, Avenue Vinet 30, 1004 Lausanne, Switzerland; 2 Community psychiatry service, Department of Psychiatry, University Hospital Center, Lausanne, Switzerland; 3 SISP SA, Lausanne, Switzerland; 4 HorizonSud, Marsens, Switzerland; 5 Fondation Pro-Home, Rolle, Switzerland; 6 Institut Universitaire de Formation et de Recherche en Soins Infirmiers, University of Lausanne, Lausanne, Switzerland

**Keywords:** Schizophrenia, Anhedonia, Apathy, Negative symptoms, Psychosocial treatment, Depression

## Abstract

**Background:**

Recent literature has distinguished the negative symptoms associated with a diminished capacity to experience (apathy, anhedonia) from symptoms associated with a limited capacity for expression (emotional blunting, alogia). The apathy-anhedonia syndrome tends to be associated with a poorer prognosis than the symptoms related to diminished expression. The efficacy of drug-based treatments and psychological interventions for these symptoms in schizophrenia remains limited. There is a clear clinical need for new treatments.

**Methods:**

This pilot study tested the feasibility of a program to reduce anhedonia and apathy in schizophrenia and assessed its impact on 37 participants meeting the ICD-10 criteria for schizophrenia or schizoaffective disorders. Participants were pre- and post-tested using the Scale for the Assessment of Negative Symptoms (SANS) and the Calgary Depression Scale for Schizophrenia (CDSS). They took part in eight sessions of the Positive Emotions Program for Schizophrenia (PEPS)—an intervention that teaches participants skills to help overcome defeatist thinking and to increase the anticipation and maintenance of positive emotions.

**Results:**

Thirty-one participants completed the program; those who dropped out did not differ from completers. Participation in the program was accompanied by statistically significant reductions in the total scores for Avolition-Apathy and Anhedonia-Asociality on the SANS, with moderate effect sizes. Furthermore, there was a statistically significant reduction of depression on the CDSS, with a large effect size. Emotional blunting and alogia remain stable during the intervention.

**Discussion:**

Findings indicate that PEPS is both a feasible intervention and is associated with an apparently specific reduction of anhedonia and apathy. However, these findings are limited by the absence of control group and the fact that the rater was not blind to the treatment objectives.

**Conclusions:**

PEPS is a promising intervention to improve anhedonia and apathy which need to be tested further in a controlled study.

**Trial registration number:**

ISRCTN registry ISRCTN74048461, registered 18 may 2015

## Background

Anhedonia—the difficulty of anticipating or experiencing pleasure—is a particularly challenging negative symptom of schizophrenia. Anhedonia is often associated with apathy and social withdrawal, and is one of schizophrenia’s characteristic features. The literature distinguishes the negative symptoms associated with a diminished capacity to experience (anhedonia, asociality, and avolition) from those which are associated with a limited capacity for expression (emotional blunting, alogia) [1, 2]. These negative symptoms can significantly contribute to a decrease in social [3] and professional [4] functioning, and they are linked to a lower quality of life [5]. The syndrome of apathy-anhedonia tends to be associated with a poorer prognosis than the symptoms related to diminished expression, which suggests that it is the more severe facet of the psychopathology [6]. The efficacy of drug-based treatments and psychological interventions remains limited [7, 8], and there is a clear clinical need for new treatments for negative symptoms [9]. In order to improve social functioning, experts strongly recommend combining psychosocial interventions with pharmacological treatments [10]. Existing psychosocial treatments, however, are not specific enough, and often only improve negative symptoms indirectly [11]. Interventions which challenge defeatist thinking do play a beneficial role [12, 13].

Several studies have underlined that individuals with schizophrenia are less active or less involved in enjoyable and positive activities than non-sufferers. Laboratory studies measuring what sufferers actually experience when confronted with pleasurable stimuli, have shown however, that patients do not experience less pleasure than control subjects [14]. This paradox is explained by the fact that individuals with schizophrenia experience a deficit of anticipatory pleasure rather than in-the-moment pleasure [15–17]. Together, patients may have an impaired ability to envision the future and this difficulty is associated with apathy [18]. These results suggest that within the syndrome of diminished capacity to experience, apathy and anhedonia, might be the results of the same underlying process: that is, a diminished capacity to anticipate a particular experience or the achievement of a pleasurable goal [19] or a motivational impairment [20]. Patients also reported lower levels of pleasure in savoring past, present, and future events than did normal controls, and stated that they had low expectations of their self-efficacy [21]. Savoring is the capacity to wait for, appreciate, and enhance a positive experience [22]. Individuals with schizophrenia also manifest a lesser ability to maintain positive emotions [23–25]. The facial expression of emotions can play a causal role in the subjective experience of those emotions [26]. Even though observable outward signs of emotional expression are lessened in schizophrenia, studies indicate that sufferers continue to display very subtle facial muscle movements (as measured by electromyogram) that are similar to and in accordance with their responses [27]. These data highlight target deficits in the temporal experience of pleasure, anticipating, maintaining and expressing positive emotions. Strauss [28] suggested maximizing positive emotional experiences by using techniques developed in the field of affective science [22, 26] to increase the frequency and duration of positive emotional experiences. Five techniques have been found to specifically and reliably increase the frequency, intensity, and duration of positive emotions. They include anticipating the enjoyment; behavioral display (expressing emotions via nonverbal behaviors); being ‘in the moment’ (directing controlled attention toward positive experiences when they occur—savoring); communicating and celebrating positive experiences with others; and recalling previously pleasurable events.

Cognitive behavior therapy (CBT) for psychosis or metacognitive training (MCT) have been shown to be effective on negative symptoms or self-esteem [29, 30]. However very few studies on schizophrenia have specifically targeted anhedonia and apathy. An open pilot trial using Loving Kindness Meditation, as a method of increasing positive emotions, led to an improvement in negative symptoms [31]. Another pilot study [32] showed that training in anticipatory pleasure led to an improvement of anhedonia and an increase in activity. PEPS (Positive Emotions Program for Schizophrenia) is a new intervention meant to improve apathy and anhedonia by increasing cognitive control of positive emotions, including the anticipation and maintenance of those emotions [33,34]. Despite the similar format of MCT and PEPS, MCT focuses mainly on delusional symptoms whilst PEPS focuses more on negative symptoms.

This pilot study’s goal was to test the program’s feasibility and its effects on individuals with a schizophrenia-spectrum disorder who presented symptoms of anhedonia. Our hypothesis was that eight sessions of PEPS would reduce anhedonia and apathy, as measured using the Scale for the Assessment of Negative Symptoms (SANS).

## Methods

This pilot study uses an open pre-post comparison within subject design.

### Participants and inclusion criteria

Participants were recruited at three social and nursing homes, in Lausanne, Rolle and Marsens—towns in French-speaking Switzerland. The inclusion criteria were:

- Fulfilling the ICD-10 criteria for a diagnosis of schizophrenia or a schizoaffective disorder.
- Presenting a score of at least 2 on the overall SANS anhedonia scale.
- Being aged between 18 and 65 years old.
- Being able to read and understand French.
- Demonstrating capacity for consent according to the *San Diego Brief Assessment of Capacity to Consent* [35]. This tool measures a patient's understanding of an information sheet. If the potential participant is unable to respond correctly to the questions asked after reading the sheet, the patient is excluded. The procedure can be conducted a maximum of twice.

### Measures

The following data and scales were used in pre- and post-tests as part of standardized interviews with a psychologist trained in their administration. The average time needed to complete the scales with the participants was one hour.

- ▪ Collection of socio-demographic and clinical data: age, sex, psychiatric diagnosis, duration of illness, living arrangements (e.g., nursing home, with family), treatment, etc.
- ▪ *Scale for the Assessment of Negative Symptoms* (SANS) [36] : this scale measures schizophrenia’s deficit symptoms within the framework of schizophrenic disorders. It comprises of 25 items, scored from 0 to 5. A definition of each item, including examples, facilitates a better understanding of scale’s content. The rating system is ordinal, from 0 (absent) to 5 (severe). The 25 items are grouped into five components: (1) withdrawal or emotional poverty; (2) alogia (lack of speech); (3) avolition and apathy (lack of energy, lack of initiative); (4) anhedonia and social withdrawal (loss of interests); (5) attention. A score is given to each component. Results can be expressed in several forms: an overall score (0 to 125), the sub-scores of each component, sum of all sub-scores or sum of the five items of the overall evaluation. The scale was translated into French with acceptable validity [37, 38].
- ▪ The Calgary Depression Scale for Schizophrenia (CDSS) [39] includes nine items: depression, hopelessness, self-depreciation, guilty ideas of reference, pathological guilt, morning depression, early wakening, suicide and observed depression. This scale has been validated in French [40].
- ▪ The Savoring Belief Inventory (SBI) is a scale for measuring beliefs about one's capacity for savoring things. It is a 24-item scale including a positive scale (12 items) and a negative scale (12 items). The scale has good validity and a high test-re-test reliability [41]. The scale measures a person's thinking regarding their capacity to savor positive experiences, in terms of past experiences, current experiences, and future anticipation.

### Intervention

PEPS is an intervention meant to reduce anhedonia and apathy. The program teaches skills to help overcome defeatist thinking and to increase the anticipation and maintenance of positive emotions. PEPS involves 8 one-hour group sessions, administered using visual and audio materials and presented as PowerPoint presentation slides projected onto a screen. The program was conceived by Jérôme Favrod and Alexandra Nguyen. The future group leaders participated in a training day and worked through all of the program's exercises together. The trainee group leaders included four nurses, two nursing assistants and four social workers—eight men and two women. Their mean age was 34.10 years (S.D. 7.45) and they had been working for an average of 9.40 years (SD 5.78) since their receiving their professional diplomas.

### Description of PEPS

A typical PEPS’s session includes the following steps. The first part of the session begins with a welcome, followed by a five minute relaxation-meditation exercise. The second part of the session starts with the group leaders going over the homework task that was given during the previous session. The session continues with an exercise in challenging specific defeatist thoughts which are presented using the program’s two fictitious heroes—Jill and Jack. Jill, for example, expresses the defeatist thinking: “I can’t relax; I’m useless.” The participant’s role is to challenge her belief, first by assigning different reasons to Jill’s difficulty to relax. They learn to find reasons which might be linked to the program’s hero, the other people or to Jill’s environment. They subsequently try to develop an alternative, a more positive way of thinking. Modifying defeatist thinking appear to be an essential supplementary ingredient to treat negative symptom because of repeated failures in patients account [13, 42, 43]. Following this introduction, and according to the session theme, participants learn and practice a new skill to improve their anticipation or maintenance of pleasure. The session ends with the setting of the homework task that the participants must accomplish for the next session.

### Key targets

The skills taught are: savoring a present or past pleasant experience, expressing emotions by increasing behavioral expression, making the most of or capitalizing on positive moments, and anticipating pleasant moments. Savoring an agreeable experience involves becoming aware of that pleasure or of the positive emotions the participant is feeling at a given moment [22]. For example, participants are invited to look at a picture of pleasant countryside or listen to soothing music, and to become aware of the pleasurable experience of doing this and to appreciate it. Increasing behavioral expression of emotions involves using facial expressions or gestures to accompany that positive emotion. The participants are invited to imitate pictures of actors expressing a positive emotion and to become aware of the sensations this produces. Making the most of positive moments entails communicating and celebrating positive events with others. For example, participants were invited to describe positive events to one another through role-play. Anticipating pleasant moments involves imagining the sensations produced by a positive future event. This strategy is meant to guide the participants through different positive feelings and emotions. It can engage their different senses, for example, by imagining they are eating a fruit, or by anticipating the emotion produced and the physical sensations experienced upon the completion of a pleasurable physical or social activity. A simple homework task is assigned to be done between each session. For example, this could be, choosing an image or an object that provokes a positive emotion or feeling in the participant, who must then bring it back and to present it to the group.

### Psychoeducational concept

The psychoeducational concept underpinning the program was built according to Kolb & Kolb's model [44] of experiential learning. Each learning activity involves: 1) concrete experience, during which the participant completes a concrete task (e.g. savoring the pleasure of looking at a beautiful landscape); 2) reflective observation, during which the participant reflects on his/her experience, his/her past—the participant communicates about the completion of the task (e.g. sharing what pleasure was provoked by looking at the landscape and how it has been savored); 3) abstract conceptualization, during which the participant interprets events—theoretical links are created or introduced by a third party (e.g. explaining the importance of savoring in daily life); and 4) active experimentation, during which the participant anticipates a new means of trying out the task, in light of the skills acquired in the preceding phase, and then executes them (e.g. experimenting savoring in daily life and taking note in a journal of the experience of savoring pleasurable experience). The program uses a collaborative, egalitarian approach. Group facilitators participate in sessions just like the participants, by doing the exercises, by sharing their experiences, and by carrying out the given tasks.

Patients participated in eight one-hour sessions of PEPS at a rate of one per week.

Session List:

- Session 1: defeatist thinking
- Session 2: savoring pleasant moments (I)
- Session 3: accentuating the behavioral expression of emotions
- Session 4: making the most of pleasant moments by sharing them with others
- Session 5: savoring past pleasant moments (II)
- Session 6: anticipating pleasant moments (I)
- Session 7: anticipating pleasant moments (II)
- Session 8: review of all skills.

Participants conduct the reviewing exercises themselves during the last session.

### Ethical aspects

Protocol 127/14, Pilot Study for the Positive Emotions Program for Schizophrenia (PEPS), a study for improving anhedonia, was accepted by the Vaud Cantonal Ethics Commission on Human Research on 6^th^ May 2014. Participants signed an informed consent to be included.

### Data analyses

Results are presented using descriptive statistics and independent and paired t-tests according to the comparisons between or within participants. Effect sizes were calculated using paired t-tests statistics, using the formula reported by Borenstein and taking into account the dependence between data points [45]. A Bonferroni correction was applied for multiple testing.

## Results and discussion

### Participants’ description

**Figure 1:**
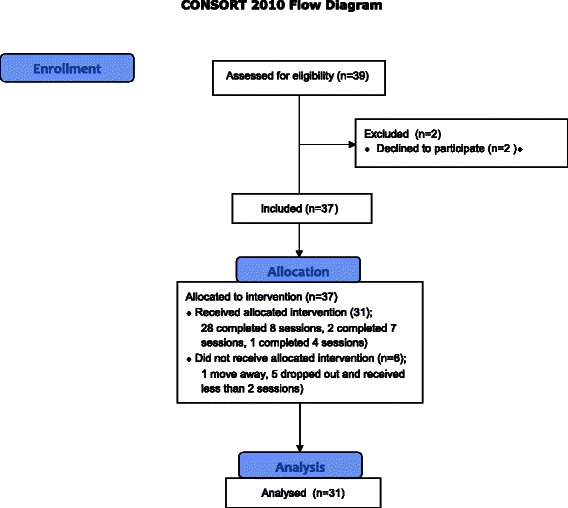
CONSORT 2010 Flow Diagram

Thirty-nine participants were recruited, two of whom refused to give their consent for the study. The final group was composed of 24 men and 13 women, with an average age of 41.84 years old (S.D. = 11.99). Thirty-one met the criteria for ICD-10 schizophrenia and six met the criteria for a schizoaffective disorder. Their mean duration of illness was 19.05 years (S.D. 12.85). Twenty-eight were single, eight were separated or divorced, and one was a widower. In terms of the educational level which they had achieved: six had not finished their mandatory schooling, 17 participants had finished their mandatory schooling, three had a secondary school diploma, eight had completed a professional apprenticeship and three had either a professional school or university diploma. Three participants lived independently, two lived with their families, and 32 lived in sheltered housing. None of the participants had a job on the competitive labor market. All participants except one were on antipsychotic medication. Fourteen were on antidepressant at mean fluoxetine equivalents of 29.60 mg (SD 14.01) [46]. Figure 1 present the CONSORT flowchart of the study. Twenty-eight participants completed all eight sessions, two completed seven sessions, and one participant in the four cohorts only completed four sessions. Six participants dropped-out, one refused to participate after the first assessment, one moved away, and four left the program before it finished. Two refused to continue the program, without giving an explanation according to the informed consent form. However, four provided reasons for giving up which were unconnected with the program (external stressors).Table 1 shows that participants who dropped out did not differ from those who completed the program. Participants who dropped out did not differ, either from those who completed PEPS on the SANS and CDSS at baseline. Participants were recruited between May 20^th^ 2014 and November 30^th^ 2015.

**Table 1:** Participant descriptions: completers vs drop-outs

	Completers	Drop-outs	Statistics test*; p-value
	*n* = 31	*n* = 6	
	Mean (S.D.) *N* (%)	Mean (S.D.) *N* (%)	
*Socio-demographic characteristics*			
Sex female/male	12/19	1/5	Fisher’s exact test; p = .39
Age	42.97 (S.D. 12.33)	36.00 (S.D. 8.67)	Mann–Whitney U = 63; p = .23
Professional/university education level	9 (29 %)	2 (33 %)	Fisher’s exact test; p =1.00
Home situation: Nursing home	24 (77 %)	5 (83 %)	Fisher’s exact test; p = 1.00
*ICD-10 diagnosis*			
Schizophrenia	27	4	Fisher’s exact test; p = .25
Schizoaffective disorders	4	2	
Duration of illness since first hospitalization (years)	19.71 (S.D. 12.69)	15.67 (S.D. 14.35)	Mann–Whitney U = 76; p = .51
*Current treatment*			
Atypical antipsychotic	24 (80 %)	6 (100 %)	Fisher’s exact test; p = .56
Typical antipsychotic	6	-	
No antipsychotic	1	-	
Antidepressant	13	1	Fisher’s exact test; p = .38
Antidepressant in fluoxetine equivalents	28.45 (13.97)	44.44	

### Comparison between pre- and post-tests.

In post-tests, the participants had significantly improved (reduced) the average total scores for the Avolition-Apathy and Anhedonia-Asociality scales of the SANS. They exhibited Cohen's *d* effect sizes of 0.50 for the Anhedonia-Asociality scale and 0.57 for the Avolition-Apathy scale, resulting in a moderate overall effect size. The average total score for the CDSS also showed a significant improvement in the post-test, with a Cohen's *d* effect size of 0.91. The affective flattening or blunting, the alogia and the attention scales of the SANS did not change during the intervention.

Finally, the average total score on the SANS and the average total score on the Savoring Belief Inventory showed small effect sizes, but they were not statistically significant because the Bonferroni correction needs a p < .001. Table 2 present the pre- and post-test results (see Table 2).

**Table 2:** Pre and post intervention differences

	Pre-test Mean (sd)	Post-test Mean (sd)	t (df); p	Cohen s’d
*SANS scale scores*				
Affective flattening or blunting	9.74 (7.43)	9.45 (5.93)	t = .24 (30); p = .82	0.04
Alogia	4.58 (2.87)	4.35 (2.22)	t = .59 (30); p = .56	0.08
Avolition – Apathy*	6.29 (3.82)	4.29 (2.76)	t = 3.84 (30); p = .001	0.57
Anhedonia – Asociality*	11.10 (3.90)	9.26 (3.26)	t = 3.81 (30); p = .001	0.50
Attention	3.42 (2.38)	2.87 (2.73)	t = 1.10 (30); p = .28	0.21
SANS-total score	35.13 (13.70)	30.23 (12.25)	t = 2.48 (30); p = .02	0.38
CDSS total score*	8.39 (4.58)	4.55 (3.72)	t = 4.61 (30); p = .000	0.91
*Savoring Belief Inventory total score*	4.52 (7.81)	7.81 (8.67)	t = −2.26 (30); p = .03	0.34

*p < .005, Boneferroni correction for multiple tests (8 comparisons)

### Secondary analyses

In order to disentangle apathy and anhedonia from depression, our sample was divided into 17 depressed and 14 not depressed participants: those with a CDSS score > 6 were classed as depressed [39, 47]. Depressed and not depressed participants were compared by using the pre-post differences in their scores for the SANS Avolition–Apathy and SANS Anhedonia–Asociality. Mann–Whitney U tests were 147.0 for the Avolition–Apathy scale(median difference for depressed participants = −1.0, semi-interquartile range = −.5; median difference for non-depressed participants = −1.0, semi-interquartile range = −1, p = .28) and 65.0 for the Anhedonia–Asociality scale (median difference for depressed participants = −3.0, semi-interquartile range = −1.5; median difference for non-depressed participants = 0.0, semi-interquartile range = −1, p = .03). The depressed participants showed more improvement than not depressed participants. At post-test, only nine of the participants were still depressed according to the > 6 cut-off point.

## Discussion

This initial study demonstrated that the Positive Emotions Program for Schizophrenia (PEPS) is a feasible treatment method. The preliminary results, presented here, indicate that PEPS was accompanied by a reduction of anhedonia, apathy, and depression in a group of participants diagnosed with schizophrenia or schizoaffective disorders. In addition, the results indicate that PEPS may have a potential specific effect on the reduced experience negative symptoms compared to the reduced expression negative symptoms. These results are encouraging and suggest the need for a randomized trial. Furthermore, the scientific results were corroborated by the patients’ enthusiasm and diligent participation, something that is not customary for this population. All the patients expressed a very positive opinion about the intervention. Nevertheless, a few participants (six) did drop out of the study (a rate of 16 %). However, a 16 % drop-out rate is only slightly higher than the average 13 % drop-out rate found in randomized clinical trials of psychosocial treatments [48]. It should be noted that the present sample corresponds to a population with a rather severe disability: their mean duration of illness was 19.05 years. Several participants spontaneously declared their appreciation of the fact that the group leaders also participated in the exercises, sharing their personal experiences and even carrying out the homework tasks. A few participants even stated that the very fact that some group leaders reported experiencing less pleasure in certain exercises than the patients did themselves, demonstrated to them that pleasure might be independent of their illness. Thus the collaborative approach alone, used as a part of the program’s psychoeducational format, may have had an impact on participants.

These results are consistent with those of two other pilot studies using ingredients of positive psychology that target positive emotions in people with schizophrenia [31, 49].

This study has several limitations. Firstly, since there was no control group, the psychologist who assessed the participants was not blind. The absence of a control group does not control for the attention given to the participants. A novelty effect is possible according to the enthusiasm of the participants who advertise to invite other patients to come to the program. Depression scores also showed improvement, and it can be difficult to distinguish depression from apathy and anhedonia. The depressed participants showed more improvement than not depressed participants, suggesting that anhedonia and depression are closely tied together. Further studies using PEPS should continue to explore these differential impacts in order to better distinguish depression from the anhedonia negative symptom in schizophrenia. Change in the Avolition–Apathy score appears to be less associated with a patient’s depression status. Since it is estimated that comorbid depression occurs in 50 % of patients with schizophrenia [50], this distinction is more conjectural than realistic. Interventions which successfully reduce depression and negative symptoms are, nevertheless, highly sought after. In further studies, it would also be interesting to examine if PEPS influence psychotic symptoms.

## Conclusions

PEPS aims at challenging defeatist thoughts and teaching new skills to anticipate and maintain positive emotions. This pilot study with PEPS was associated with a reduction in depression, apathy, and anhedonia. Affective flattening and alogia remain stable during the intervention, suggesting a potential specific effect of PEPS on anhedonia and apathy. These favorable results should be now studied further using a randomized trial.

## Availability of data and materials

Data are available from the first author, Materials is available on http://www.seretablir.net/outils-interventions/peps/.

## Abbreviations

CDSS: Calgary Depression Scale for Schizophrenia
ICD-10: International Statistical Classification of Diseases and Related Health Problems 10th Revision
PEPS: Positive Emotions Program for Schizophrenia
SANS: Scale for the Assessment of Negative Symptoms
SBI: Savoring Belief Inventory

## References

[1] Blanchard JJ, Cohen AS (2006). The structure of negative symptoms within schizophrenia: implications for assessment. Schizophr Bull.

[2] Hartmann MN, Hager OM, Reimann AV, Chumbley JR, Kirschner M, Seifritz E, Tobler PN, Kaiser S (2015). Apathy but not diminished expression in schizophrenia is associated with discounting of monetary rewards by physical effort. Schizophr Bull.

[3] Ventura J, Wood RC, Hellemann GS (2013). Symptom domains and neurocognitive functioning can help differentiate social cognitive processes in schizophrenia: a meta-analysis. Schizophr Bull.

[4] Tsang HW, Leung AY, Chung RC, Bell M, Cheung WM (2010). Review on vocational predictors: a systematic review of predictors of vocational outcomes among individuals with schizophrenia: an update since 1998. Aust N Z J Psychiatry.

[5] Eack SM, Newhill CE (2007). Psychiatric symptoms and quality of life in schizophrenia: a meta-analysis. Schizophr Bull.

[6] Strauss GP, Horan WP, Kirkpatrick B, Fischer BA, Keller WR, Miski P, Buchanan RW, Green MF, Carpenter WT (2013). Deconstructing negative symptoms of schizophrenia: avolition-apathy and diminished expression clusters predict clinical presentation and functional outcome. J Psychiatr Res.

[7] Turkington D, Morrison AP (2012). Cognitive therapy for negative symptoms of schizophrenia. Arch Gen Psychiatry.

[8] Kendall T (2012). Treating negative symptoms of schizophrenia, vol. 344.

[9] Fusar-Poli P, Papanastasiou E, Stahl D, Rocchetti M, Carpenter W, Shergill S, McGuire P (2014). Treatments of Negative Symptoms in Schizophrenia: Meta-Analysis of 168 Randomized Placebo-Controlled Trials. Schizophr Bull.

[10] Swartz MS, Perkins DO, Stroup TS, Davis SM, Capuano G, Rosenheck RA, Reimherr F, McGee MF, Keefe RS, McEvoy JP (2007). Effects of antipsychotic medications on psychosocial functioning in patients with chronic schizophrenia: findings from the NIMH CATIE study. Am J Psychiatry.

[11] Elis O, Caponigro JM, Kring AM (2013). Psychosocial treatments for negative symptoms in schizophrenia: current practices and future directions. Clin Psychol Rev.

[12] Granholm E, Holden J, Link PC, McQuaid JR, Jeste DV (2013). Randomized controlled trial of cognitive behavioral social skills training for older consumers with schizophrenia: defeatist performance attitudes and functional outcome. Am J Geriatr Psychiatry.

[13] Couture SM, Blanchard JJ, Bennett ME (2011). Negative expectancy appraisals and defeatist performance beliefs and negative symptoms of schizophrenia. Psychiatry Res.

[14] Kring AM, Caponigro JM (2010). Emotion in Schizophrenia: Where Feeling Meets Thinking. Curr Dir Psychol Sci.

[15] Buck B, Lysaker PH (2013). Consummatory and anticipatory anhedonia in schizophrenia: stability, and associations with emotional distress and social function over six months. Psychiatry Res.

[16] Favrod J, Ernst F, Giuliani F, Bonsack C (2009). Validation française de l'échelle d'expérience temporelle du plaisir. Encéphale.

[17] Gard DE, Kring AM, Gard MG, Horan WP, Green MF (2007). Anhedonia in schizophrenia: distinctions between anticipatory and consummatory pleasure. Schizophr Res.

[18] Raffard S, Esposito F, Boulenger JP, Van der Linden M (2013). Impaired ability to imagine future pleasant events is associated with apathy in schizophrenia. Psychiatry Res.

[19] Foussias G, Remington G (2010). Negative symptoms in schizophrenia: avolition and Occam's razor. Schizophr Bull.

[20] Strauss GP, Morra LF, Sullivan SK, Gold JM (2014). The Role of Low Cognitive Effort and Negative Symptoms in Neuropsychological Impairment in Schizophrenia. Neuropsychology.

[21] Cassar R, Applegate E, Bentall RP (2013). Poor savouring and low self-efficacy are predictors of anhedonia in patients with schizophrenia spectrum disorders. Psychiatry Res.

[22] Bryant FB (2007). The Process of Savoring: A New Model of Positive Experience.

[23] Ursu S, Kring AM, Gard MG, Minzenberg MJ, Yoon JH, Ragland JD, Solomon M, Carter CS (2011). Prefrontal cortical deficits and impaired cognition-emotion interactions in schizophrenia. Am J Psychiatry.

[24] Kring AM, Germans Gard M, Gard DE (2011). Emotion deficits in schizophrenia: timing matters. J Abnorm Psychol.

[25] Horan WP, Wynn JK, Kring AM, Simons RF, Green MF (2010). Electrophysiological correlates of emotional responding in schizophrenia. J Abnorm Psychol.

[26] Quoidbach J, Berry EV, Hansenne M, Mikolajczak M (2010). Positive emotion regulation and well-being: Comparing the impact of eight savoring and dampening strategies. Personality and Individual Differ.

[27] Kring AM, Moran EK (2008). Emotional response deficits in schizophrenia: insights from affective science. Schizophr Bull.

[28] Strauss GP (2013). Translating basic emotion research into novel psychosocial interventions for anhedonia. Schizophr Bull.

[29] Moritz S, Veckenstedt R, Andreou C, Bohn F, Hottenrott B, Leighton L, Kother U, Woodward TS, Treszl A, Menon M (2014). Sustained and "sleeper" effects of group metacognitive training for schizophrenia: a randomized clinical trial. JAMA Psychiat.

[30] Turkington D, Sensky T, Scott J, Barnes TR, Nur U, Siddle R, Hammond K, Samarasekara N, Kingdon D (2008). A randomized controlled trial of cognitive-behavior therapy for persistent symptoms in schizophrenia: a five-year follow-up. Schizophr Res.

[31] Johnson DP, Penn DL, Fredrickson BL, Kring AM, Meyer PS, Catalino LI, Brantley M (2011). A pilot study of loving-kindness meditation for the negative symptoms of schizophrenia. Schizophr Res.

[32] Favrod J, Giuliani F, Ernst F, Bonsack C (2010). Anticipatory pleasure skills training: a new intervention to reduce anhedonia in schizophrenia. Perspect Psychiatr Care.

[33] Kring AM, Elis O (2013). Emotion deficits in people with schizophrenia. Annu Rev Clin Psychol.

[34] Favrod J, Rexhaj S, Nguyen A, Cungi C, Bonsack C, Ritsner MS (2014). Projecting Oneself into the Future, an Intervention for Improving Pleasure in Patients with Anhedonia. Anhedonia: A Comprehensive Handbook Volume I: Conceptual Issues And Neurobiological Advances. Volume 1.

[35] Jeste DV, Palmer BW, Appelbaum PS, Golshan S, Glorioso D, Dunn LB, Kim K, Meeks T, Kraemer HC (2007). A new brief instrument for assessing decisional capacity for clinical research. Arch Gen Psychiatry.

[36] Lecrubier Y, Boyer P (1987). L'utilisation de la SANS et de la SAPS. Psychiatr Psychobiol.

[37] Dollfus S, Langlois S, Assouly-Besse F, Petit M (1995). Depressive symptoms and negative symptoms during schizophrenia]. Encéphale.

[38] Lecrubier Y, Boyer P (1987). Fiche descriptive et traduction française de la SANS. Psychiatrie & Psychobiologie.

[39] Addington D, Addington J, Maticka-Tyndale E (1993). Assessing depression in schizophrenia: the Calgary Depression Scale. Br J Psychiatry Suppl.

[40] Reine G, Bernard D, Auquier P, Le Fur B, Lancon C (2000). [Psychometric properties of French version of the Calgary depression scale for schizophrenics (CDSS)]. Encéphale.

[41] Bryant FB (2003). Savoring Beliefs Inventory (SBI): A scale for measuring beliefs about savouring. J Ment Health.

[42] Rector NA, Beck AT, Stolar N (2005). The negative symptoms of schizophrenia: a cognitive perspective. Can J Psychiat Rev canadienne de psychiatrie.

[43] Grant PM, Beck AT (2009). Defeatist beliefs as a mediator of cognitive impairment, negative symptoms, and functioning in schizophrenia. Schizophr Bull.

[44] Kolb AY, Kolb DA (2008). The Learning Way: Meta-cognitive Aspects of Experiential Learning. Simulation & Gaming.

[45] Borenstein M, Cooper H, Hedges LV, Valentine JC (2009). Effect sizes for continuous data. *The handbook of research synthesis and meta analysis* edn.

[46] Hayasaka Y, Purgato M, Magni LR, Ogawa Y, Takeshima N, Cipriani A, Barbui C, Leucht S, Furukawa TA (2015). Dose equivalents of antidepressants: Evidence-based recommendations from randomized controlled trials. J Affect Disord.

[47] Schennach R, Obermeier M, Seemuller F, Jager M, Schmauss M, Laux G, Pfeiffer H, Naber D, Schmidt LG, Gaebel W (2012). Evaluating depressive symptoms in schizophrenia: a psychometric comparison of the Calgary Depression Scale for Schizophrenia and the Hamilton Depression Rating Scale. Psychopathology.

[48] Villeneuve K, Potvin S, Lesage A, Nicole L (2010). Meta-analysis of rates of drop-out from psychosocial treatment among persons with schizophrenia spectrum disorder. Schizophr Res.

[49] Meyer PS, Johnson DP, Parks A, Iwanski C, Penn DL (2012). Positive living: A pilot study of group positive psychotherapy for people with schizophrenia. J Positive Psychol.

[50] Buckley PF, Miller BJ, Lehrer DS, Castle DJ (2009). Psychiatric comorbidities and schizophrenia. Schizophr Bull.

